# Optical coherence tomography assessment of axonal and neuronal damage of the retina in patients with familial and sporadic multiple sclerosis

**DOI:** 10.3389/fneur.2022.953188

**Published:** 2022-09-16

**Authors:** Monika Grudziecka Pyrek, Krzysztof Selmaj

**Affiliations:** ^1^Department of Neurology, University of Warmia and Mazury, Olsztyn, Poland; ^2^Centrum of Neurology, Lodz, Poland

**Keywords:** familial multiple sclerosis, sporadic multiple sclerosis, retinal nerve fiber layer, macular volume, ganglion cell and inner plexiform layer, inner nuclear layer, optical coherence tomogaphy

## Abstract

**Objective:**

To assess axonal and neuronal damage of the retina in patients with familial (fMS) and sporadic multiple sclerosis (sMS).

**Methods:**

87 relapsing-remitting MS patients (45 patients with sMS, 42 patients with fMS) and 30 healthy controls were included in the study. Optical coherence tomography (OCT) was performed with the spectral domain optical coherence tomography (SD-OCT, Heidelberg Engineering, Germany). The peripapillary retinal nerve fiber layer (pRNFL) thickness, ganglion cell-inner plexiform layer (GCIPL) thickness, total macular volume (TMV) and the inner nuclear layer (INL) thickness were measured.

**Results:**

A significant reduction of the pRNFL thickness was detected in sMS and fMS compared to the control group (86.29 (+/- 16.13) μm in sMS, 84.78 (+/- 12.92) μm in fMS, 98.93 (+/- 6.71) μm in control group; *p* < 0.001). There was no significant difference in the pRNFL thickness between sMS and fMS (*p* = 0.5239). The GCIPL thickness was significantly decreased in sMS and fMS compared to the control group [66.0581 (+/- 11.2674) μm in sMS, 63.8386 (+/-10.004) μm in fMS, 76.5074 (+/- 5.0004) μm in control group; *p* < 0.001]. A significant reduction of the TMV was shown in sMS and fMS compared to the control group [8.4541(+/- 0.4727) mm3 in sMS, 8.3612 (+/- 0.4448) mm3 in fMS, 8.8387 (+/- 0.314) mm3 in control group; *p* < 0.0011]. No difference in the GCIPL thickness and TMV between sMS and fMS was found (*p* = 0.3689 and *p* = 0.3758, respectively). The INL thickness in sMS and fMS did not differ compared to the control group [34.2323 (+/- 2.7006) μm in sMS, 34.5159 (+/- 2.9780) μm in fMS, 33.6148 (+/- 2.0811) μm in control group; *p* = 0.5971 and *p* = 0.1870, respectively] and between the two forms (*p* = 0.4894).

**Conclusion:**

We confirmed the presence of axonal and neuronal damage of the retina in sMS and fMS. Both forms of MS did not differ significantly from each other with respect to RFNL, GCIPL, MV and INL. ON induced significant reduction of the pRNFL, GCIPL and MV in both groups of pwMS.

## Introduction

Multiple sclerosis (MS) is the most common chronic inflammatory demyelinating disorder of the central nervous system (CNS). Two major forms of MS have been recognized: sporadic (sMS) and familial (fMS). In fMS at least two family members should be diagnosed with MS (1). It is estimated that fMS affects between 12.6 and 20% of all patients with MS (pwMS) (2–4). The genetics of MS is complex and correspond to polygenic trait. It is believed that MS genetics can explain up to half of the disease's heritability (5, 6). Classical twin studies showed that unaffected monozygotic twin have much higher risk to develop MS, 25–30%, than dizygotic twin, 2-5% (7, 8). The average risk of developing MS by relatives of pwMS ranged from 3 to 5%, with the highest risk for first-degree relatives, and was 30 to 50 times higher than the 0.1% risk for the general population (2). These data suggest that fMS might represent a more genetically driven form of MS versus sMS. Clinical phenotyping of these two forms of MS did not reveal any major differences (9–11). However, some discordance was observed. It was found that pwMS with multiple affected relatives had a higher incidence of optic neuritis (ON) as the first relapse, a lower risk of another relapse in the first year of disease, a longer interval between the first and second relapse and a longer time to permanent neurological deficit (3). Also MRI studies have shown some differences between these two forms of MS. In fMS compared to sMS was observed a larger T1-lesion volume and a trend toward lower magnetization transfer ratio (MTR) of T1-lesions (12). Our own earlier studies showed that MTR abnormalities were more widespread in fMS than in sMS. MTR values were reduced mainly in the corpus callosum and in the cerebral and cerebellar peduncles, primarily involving areas of highly myelinated white matter (13). Proton magnetic resonance spectroscopy showed a slight decrease in N-acetylaspartate (NAA) -to-choline (Cho) and NAA-to-creatine (Cr) ratios in normal appearing white matter in sMS versus fMS, whereas Cho/Cr ratio showed an increased trend. These results might indicate more pronounced neurodegenerative injury in sMS than in fMS (14).

To address this hypothesis, we applied the optical coherence tomography (OCT) technique and aimed to study axonal and neuronal damage of the retina in patients with sMS and fMS. OCT is a non-invasive interferometric technique that *in vivo* evaluates the thickness of several layers of the retina, including the retinal ganglion cell neurons and their axons. The retinal nerve fibre layer (pRNFL) is formed by unmyelinated retinal ganglion cell axons, whereas ganglion cell neurons are a major component of the macula (15, 16). The ganglion cell and inner plexiform layer (GCIPL) thinning and total macular volume (TMV) reduction are considered as a quantitative markers of neuronal damage in the brain (17–21). Several studies reported a positive correlation of the peripapillary RNFL (pRNFL), GCIPL thinning and TMV reduction with brain atrophy in MS, which suggested that these parameters might be indirect markers of diffuse axonal and neuronal damage in the brain (22–24). In addition, recently the inner nuclear layer (INL) was associated with inflammatory changes in MS and its thickness increases due to optic neuritis (ON) (25, 26).

We analysed the pRNFL thickness, GCIPL thickness, TMV and INL thickness in a cohort of patients with fMS and sMS and healthy controls and investigated how ON affected the OCT parameters in patients with these two forms of MS.

## Materials and methods

### Study design

In this prospective study, we analysed data from patients with relapsing-remitting multiple sclerosis (pwRRMS) and healthy controls of Medical University of Lodz, Poland, between 2012 and 2016. All study participants had OCT examination with the pRNFL and macular measurements, GCIPL, GCL, IPL, INL, and MV, of both eyes. Two groups of pwRRMS patients were analysed, sMS patients and patients with fMS. To evaluate the effect of ON on OCT parameters in sMS and fMS, the examined eyes were divided into two subgroups: eyes with a history of ON and eyes without a history of ON (Non-ON). To avoid artificial increase in the sample size of the Non-ON eyes and eyes of healthy controls we analysed one random selected eye of those groups.

### Participants

A total of 103 pwRRMS and 33 healthy controls, aged 18 – 60 were screened in the study. MS diagnosis was confirmed according to the McDonald 2010 criteria (27). Patients with fMS required to have at least one more MS case within relatives of kinship degree 1 (*n* = 13), 2 (*n* = 17) and 3 (*n* = 12). Patients with fMS and sMS were randomized according to age, duration of MS, EDSS and a history of ON. After excluding patients diagnosed with ON within 6 months prior to baseline and with other ophthalmic disorders, which might affect OCT measures, patients who did not match OSCAR-Ib criteria (28) and outliners, we finally included into the study 45 patients with sMS, 42 with fMS and 30 controls.

The study was approved by Ethical Commission of the Medical University of Lodz; Ethic Approval/Registration Numbers: RNN/83/13/KE, RNN/178/16/KE. All study participants gave written informed consent to participate in the study.

### Optical coherence tomography (OCT)

The OCT scans and all clinical data were collected on the same day as baseline visit in Medical University of Lodz, Poland. OCT exam, according to the APOSTEL recommendations (29), was performed and assessed by single certified operator on the one spectral domain OCT device in a darkened room without using of pharmacological pupil dilators (Heidelberg Spectralis OCT software version 5.4.6). Visible retinal pathology at the fundus excluded from scanning. A circular pRNFL thickness scan was performed from the right and left eye, with a diameter of 12 degrees around the optic nerve disc, which in millimetres was 3.5-3.6mm for the typical axial length of the eye. The scan circle was automatically centred on the optic nerve disc and corrected manually before taking it. Automatic Real Time (ART) function with TruTrack technology was activated, 16 frames of the same scanning area were performed. Artefacts resulting from the reflection of vessels or the lack of continuity of the pRNFL were manually corrected on an ongoing basis. The OCT software automatically calculated pRNFL thickness and showed it as a circular diagram with global (360 degrees) pRNFL thickness and pRNFL thickness in 4 segments: nasal, temporal, superior and inferior (each 90 degrees) and in the papillomacular bundle (PMB). The obtained results were comparable to the normative database of age, race, and gender, uploaded to the device, giving a colour image of the pRNFL thickness. The macula area was scanned in 25 sections in 240 μm inter-scan distance. A 6-mm diameter circular macular volume scan was performed with the centre manually located in the fovea. The segmentation algorithm was used to calculate GCL, IPL and INL thickness. The quality of the images was assessed by the same operator, based on the OSCAR-IB criteria. For the further analysis, were included scans with signal strength more than 15, clear fundus image with a sharp vascular drawing, clear border of the optic nerve disc, images of the retinal cross-section with a continuous layers of the retina and macular volume scans without blank areas, scans centred on the optic head nerve or fovea, respectively.

### Expanded disability status scale (EDSS)

Study participants were neurologically examined and their disability was assessed according to the Expanded Disability Status Scale (EDSS), which includes 8 functional systems: pyramidal, cerebellar, brainstem, sensory, bowel and bladder, visual, cerebral and other neurologic findings attributed to MS (30). In each functional system, the patients received a certain point value from which the final EDSS score was then calculated.

### Statistical analysis

To compare age of the study participants the analysis of variance (ANOVA) method was used. Student's t test was used to compare differences in duration of MS and time since ON and Kruskal-Wallis test was used to compare EDSS. Percentage of patients with a history of ON was compared using Chi-squared test. Shapiro-Wilk and D'Agostino-Pearson tests were used to assess the normality of distributions. Student's t test and Mann-Whitney test (after Fisher-Snedecor test) were used to compare OCT parameters between both groups of pwRRMS and healthy controls. ANOVA method was used to compare multiple groups with *post hoc* Student Newman Keuls test. A *p*-value of <0.05 was considered as significant. We performed all statistical analyses using the MedCalc software (V.18.2).

## Results

### Patients characteristics

After randomisation, 87 patients and 30 healthy controls were included in the study. 45 patients with sMS and 42 with fMS. Demographic and clinical data are shown in Table 1. The pw fMS and sMS did not statistically differ according to age, duration of MS, EDSS, number of relapses in the last 2 years before OCT examination. Percentage of patients with a history of ON and time since ON was similar. Number of ON in pw fMS was 32, and in sMS 25.

**Table 1:** Demographics of study participants.

	**fMS**	**sMS**	**Control**	***p* – value**
Number of participants in the study	42	45	30	
Age (+/- SD)	39.19 (9.23)	40.51(10.32)	37.07 (9.15)	0.321
Number of women	31	27	21	
Number of men	11	18	9	
Duration of MS (years) (+/- SD)	10.42 (6.792)	10.08 (7.62)	-	0.81
EDSS (IQR)	2 (2)	2 (1)	-	0.93
Number of relapses in the last 2 years before OCT examination (IQR)	1 (1)	1 (2)	-	0.38
Percentage of patients with a history of ON	52.38% (22)	42.22% (19)	-	0.35
Time since ON (years), (+/- SD)	10.29 (6.47)	10.40 (8.21)	-	0.964

fMS, familial multiple sclerosis; sMS, sporadic multiple sclerosis; ON, optic neuritis; SD, standard deviation.

### Comparison of the pRNFL thickness between sMS, fMS and control group

The pRNFL thickness in sMS and in fMS were significantly lower compared to the control group. In the segmental analysis of pRNFL, in fMS and sMS the pRNFL was thinner in all segments compared to controls, reaching the greatest differences in the papillomacular bundle (PMB), temporal and inferior segments (p<0,001). Comparative analysis of sMS with fMS showed no significant difference in the pRNFL and segmental thickness (Table 2).

**Table 2:** Comparison of the pRNFL thickness (μm) between sMS, fMS, and control group.

	**OCT parameters (**+**/-SD)**			***p*** **– value**		
	**fMS**	**sMS**	**control**	**fMS vs. sMS**	**fMS vs. control**	**sMS vs. control**
pRNFL	84.78 (12.92)	86.29 (16.13)	98.93 (6.71)	0.5239	<0.001	<0.001
pRNFL in the temporal segment	53.76 (15.26)	55.56 (16.54)	68.50 (10.16)	0.6433	<0.001	<0.001
pRNFL in the papillomacular bundle (PMB)	40.07 (11.74)	42.87 (11.84)	52.80 (7.93)	03201	<0.001	<0.001
pRNFL in the superior segment	107.92 (17.51)	111.49 (20.82)	123.18 (12.61)	0.28	0.0002	0.0125
pRNFL in the nasal segment	65.09 (15.66)	67.76 (15.69)	74.1 (10.51)	0.3908	0.0123	0.1117
pRNFL in the inferior segment	112.70 (17.18)	110.34 (17.18)	129.08 (22.73)	0.677	<0.001	<0.001

pRNFL, peripapillary retinal nerve fiber layer; fMS, familial multiple sclerosis; sMS, sporadic multiple sclerosis; SD, standard deviation.

### Comparison of the GCIPL, TMV and INL between sMS, fMS and control group

The GCIPL thickness and TMV were significantly lower in sMS and fMS patients compared to the control group and the INL thickness did not differ between the pwRRMS and the control group. There was also no significant differences in the thickness of GCIPL and INL and TMV between sMS and fMS (Table 3).

**Table 3:** Comparison of the GCIPL, GCL, IPL, INL thickness (μm) and MV (mm3) between sMS, fMS and control group.

	**OCT parameters (**+**/-SD)**			***p*** **– value**		
	**fMS**	**sMS**	**control**	**fMS vs. sMS**	**fMS vs. control**	**sMS vs. control**
GCIPL	63.8386 (10.004)	66.0581 (11.2674)	76.5074 (5.0004)	0.3689	<0.001	<0.001
GCL	33.6587 (6.1574)	35.0682 (6.5834)	41.4370 (2.9838)	0.3100	<0.0001	<0.0001
IPL	30.1799 (3.9764)	30.9899 (4.7773)	35.0704 (2.1940)	0.4419	<0.0001	0.0001
INL	34.5159 (2.9780)	34.2323 (2.7006)	33.6148 (2.0811)	0.4894	0.1870	0.5971
MV	8.3612 (0.4448)	8.4541 (0.4727)	8.8387 (0.3140)	0.3758	<0.001	0.0011

GCIPL, ganglion cell-inner plexiform layer; GCL, ganglion cell layer; IPL, inner plexiform layer; INL, inner nuclear layer; MV, macular volume; fMS, familial multiple sclerosis; sMS, sporadic multiple sclerosis; SD, standard deviation.

### Impact of ON on the pRNFL, GCIPL, TMV, and INL in sMS and fMS

All OCT parameters were measured in eyes with a history of optic neuritis (ON), sMS (*n* = 19) and fMS (*n* = 22), in eyes without a history of optic neuritis (Non-ON), sMS (*n* = 26), and fMS (*n* = 20), and in eyes from the control subjects (*n* = 30).

In ON eyes the pRNFL thickness, GCIPL thickness and TMV in sMS and fMS were significantly lower compared to control eyes. In Non-ON eyes pRNFL showed lower values in both forms of MS compared to controls, but did not reached statistical significance in sMS. In Non-ON eyes GCIPL was statistically reduced in sMS and fMS, while TMV was reduced in both forms of MS, but statistically confirmed only for fMS (Tables 4, 5).

**Table 4:** Impact of ON on the pRNFL, GCIPL, GCL, IPL, INL thickness (μm) and MV (mm3) in sMS.

**sMS**	**OCT parameters in sMS (**+**/-SD)**			***p*** **– value**		
	**Non-ON**	**ON**	**Control**	**Non-ON vs. ON**	**Non-ON vs. control**	**ON vs. control**
pRNFL	93.4231 (13.2126)	76.526 (14.8150)	98.9333 (6.7053)	0.0008	0.0563	<0.0001
GCIPL	71.7521 (8,3703)	57.8333 (9.8425)	76.5074 (5.0004)	0.0001	0.0144	<0.0001
GCL	38.3462 (4.7483)	30.3333 (6.0250)	41.4370 (2.9838)	0.0001	0.0053	<0.0001
IPL	33.4060 (3.7368)	27.5000 (3.9205)	35.0704 (2.1940)	<0.0001	0.0354	<0.0001
INL	34.0128 (2.5213)	34.5494 (2.9864)	33.6148 (2.0811)	0.7836	0.7238	0.5723
MV	8.6515 (0.4362)	8.1689 (0.3736)	8.8387 (0.3140)	0.0011	0.1224	<0.0001

pRNFL, peripapillary retinal nerve fiber layer; GCIPL, ganglion cell-inner plexiform layer; GCL, ganglion cell layer; IPL, inner plexiform layer; INL, inner nuclear layer; MV, macular volume; sMS, sporadic multiple sclerosis; Non-ON, eyes without a history of optic neuritis; ON, eyes with a history of optic neuritis; SD, standard deviation.

**Table 5:** Impact of ON on pRNFL, GCIPL, GCL, IPL, INL thickness (μm) and MV (mm3) in fMS.

**fMS**	**OCT parameters in fMS (**+**/-SD)**			***p*** **– value**		
	**Non-ON**	**ON**	**Control**	**Non-ON vs. ON**	**Non-ON vs. Control**	**ON vs. control**
pRNFL	93.0500 (9.5172)	77.2727 (10.9639)	98.9333 (6.7053)	<0.0001	0.0392	<0.0001
GCIPL	70.6056 (6.7998)	57.6869 (8.3864)	76.5074 (5.0004)	<0.0001	0.0028	<0.0001
GCL	38.0000 (3.8845)	29.7121 (5.1092)	41.4370 (2.9838)	<0.0001	0.0041	<0.0001
IPL	32.6056 (3.0143)	27.9747 (3.4573)	35.0704 (2.1940)	0.0001	0.0052	<0.0001
INL	33.5111 (2.9743)	35.4293 (2.7340)	33.6148 (2.0811)	0.0524	0.6274	0.0095
MV	8.4920 (0.3857)	8.2423 (0.4696)	8.8387 (0.3140)	0.1042	0.0023	<0.001

pRNFL, peripapillary retinal nerve fiber layer; GCIPL, ganglion cell-inner plexiform layer; GCL, ganglion cell layer; IPL, inner plexiform layer; INL, inner nuclear layer; MV, macular volume; fMS, familial multiple sclerosis; Non-ON, eyes without a history of optic neuritis; ON, eyes with a history of optic neuritis; SD, standard deviation.

The INL thickness in ON eyes was equal in both MS forms and control subjects. Similarly, in Non-ON eyes INL was not different between sMS, fMS and controls. (Tables 4, 5).

**Figure 1:**
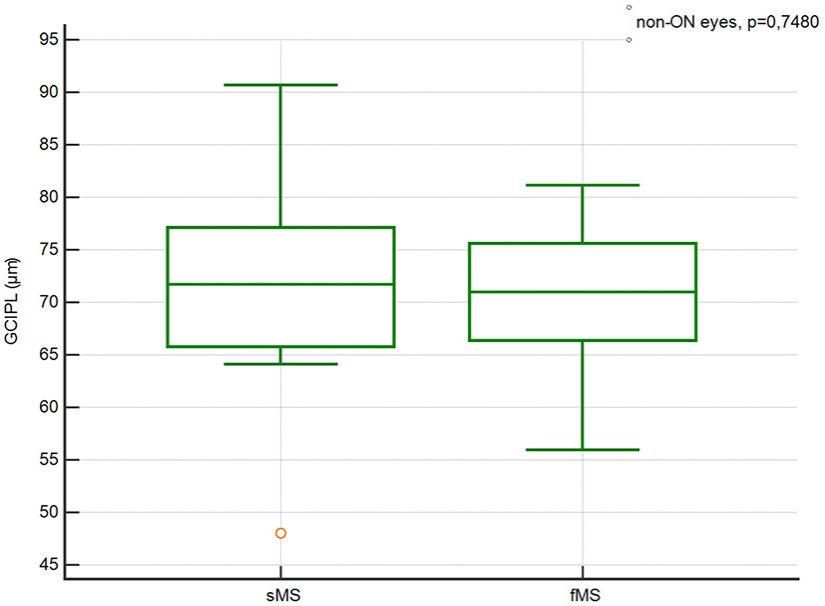
Comparison of the pRNFL thickness in Non-ON eyes between sMS and fMS.

**Figure 2:**
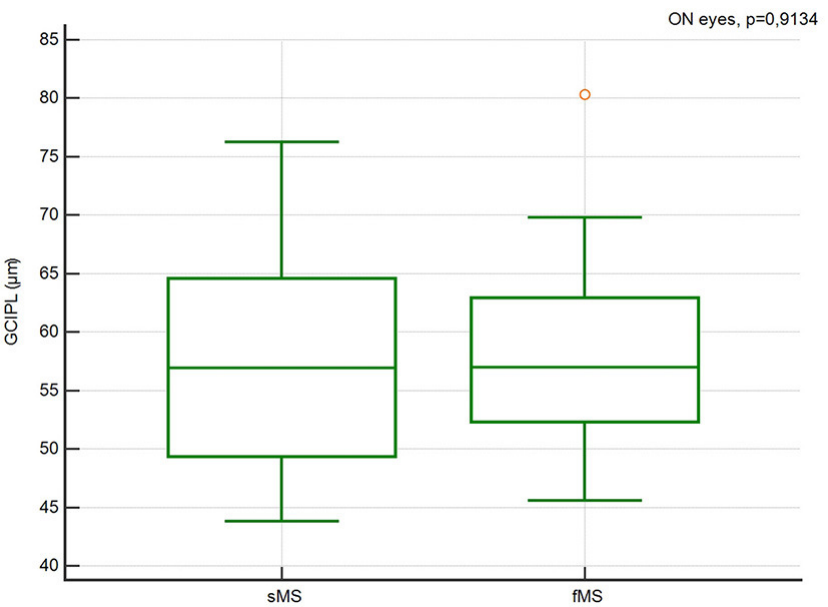
Comparison of the pRNFL thickness in ON eyes between sMS and fMS.

**Figure 3:**
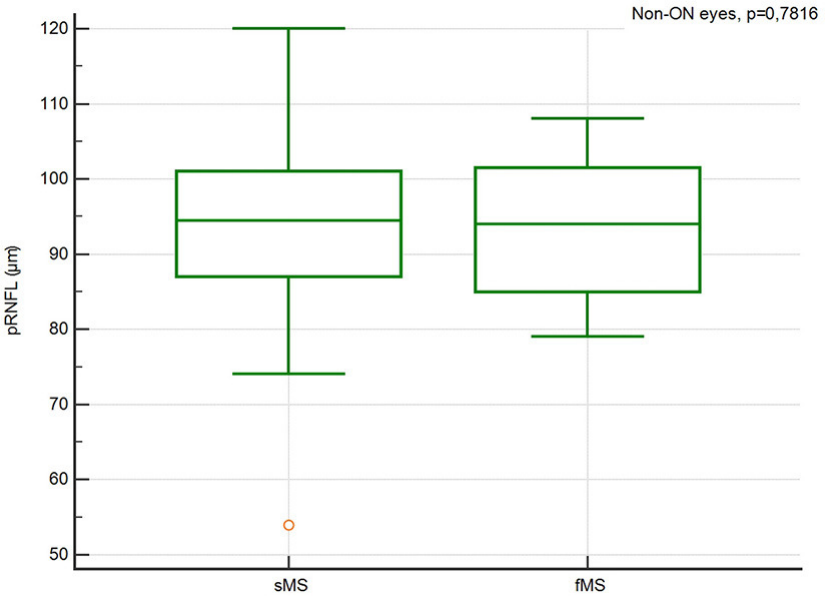
Comparison of the GCIPL thickness in Non-ON eyes between sMS and fMS.

**Figure 4:**
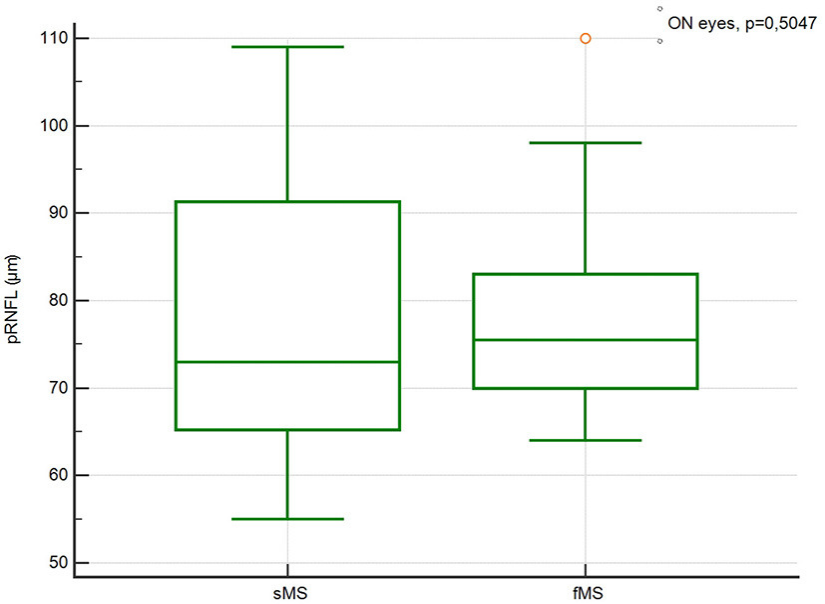
Comparison of the GCIPL thickness in ON eyes between sMS and fMS.

There were no significant differences in the pRNFL thickness, GCIPL thickness, TMV and INL thickness in ON and Non-ON eyes between sMS and fMS (Figures 1–4).

## Discussion

We have found that the pRNFL thickness, GCIPL thickness and TMV in sMS and fMS were significantly reduced in comparison to the control subjects. These findings confirmed the presence of axonal and neuronal damage of the retina in pwRRMS. However, when pRNFL and macular parameters, GCIPL and MV, were directly compared between sMS and fMS we were not able to demonstrate any significant difference. Thus, in this study retinal pathology showed similar changes in these two forms of MS.

Over the last several years numerous studies demonstrated diminished pRNFL in pwMS (31–34). This phenomenon in the classical interpretation represents a consequence of its optic nerve demyelination, leading to retrograde axonopathy as thinning of the RNFL. Most importantly, the pRNFL thickness correlated to diffuse axonal changes throughout the CNS of pwMS (23, 24). In particular pRNFL correlated with global and regional brain atrophy (17–19, 23, 24), with MRI lesion load (23, 35), MTR measures (36), with NAA/Cho ratio in proton brain MRI spectroscopy (37), with cerebrospinal fluid neurofilament light chain levels (38, 39), neurofilament light chain levels in serum (40), EDSS progression (41), cognitive decline (42) and disease-modifying treatment (DMT) response (43). In addition, similarly to pRNFL, the reduction of TMV and GCIPL thickness was also reported in pwMS and these macular measures also correlated with brain pathology and enhanced brain atrophy in pwMS (17, 19–21). Thus, optic nerve pathology in MS not only affected axonal layer represented by RNFL but also neuronal components measured by GCIPL and MV. The relative proportion of axonal to neuronal damage remains of interest and their mutual contribution to the retina pathology represent a subject of investigation. Our finding that axonal and neuronal damage of the retina is not different between sMS and fMS might suggest that the pathological mechanisms induced with optic nerve and higher optic pathways injury affecting the retina is not different in these two forms of MS.

This finding might be of interest from the perspective of still ongoing discussion on the distinction between fMS and sMS. The major issue is how much these two forms might differ in terms of genetic background leading to some differences in pathologic mechanisms. The attempt to characterize fMS by genetic screening is not available yet. However, in that context, of interest might be results of studies on MRI findings in asymptomatic relatives of patients with sMS and fMS. The 11% of asymptomatic siblings of the MS patients showed demyelinating brain lesions similar to that seen in MS (44). Our own earlier study with MTR assessment of normal appearing white matter reported diminished MTR histogram peak heights in asymptomatic relatives of MS patients (45). Similarly a study with proton magnetic resonance spectroscopy showed lower NAA/Cho and higher Cho/Cr ratio in asymptomatic relatives of pwMS (15). In the large cohort of first degree asymptomatic relatives of sMS and fMS patients a higher prevalence of MRI lesions was found in fMS, 10% compared with 4% in sMS (46). The demonstrated differences in brain imaging in relatives of patients with fMS and sMS may perhaps still support the existence of some genetic differences between these two forms of MS. On the other hand analysis of clinical presentation of sMS versus fMS did not disclose any major differences between these two forms of MS.

Our results have some obvious limitations related to the definition of fMS. However, the cohort of pwRRMS used in this study was derived from prospective analysis reducing biases related to the development of new cases in relatives over time. Also, the evaluation of the visual function was based on history of visual function impairment during ON and the history of subclinical ON was not checked. In addition the mean age of pwMS in this study, 39.19 for fMS and 40.51 years for sMS, was clearly above pooled mean age of fMS onset of 15 studies reported as 28.7 years (47). And although our finding did not demonstrate differences in the retinal pathology of familial and sporadic MS, the low sample size may be a potential source of bias and confirmation of differences in the degree of axonal and neuronal damage in both forms of MS would require further studies.

In conclusion, the results of this study showed no differences in the pRNFL and macular measures, GCIPL and MV, in pwMS with sMS and fMS. We have also not seen differences in the retinal pathology induced with ON in these two forms of disease.

## Data availability statement

The raw data supporting the conclusions of this article will be made available by the authors, without undue reservation.

## Ethics statement

The studies involving human participants were reviewed and approved by Ethical Commission of the Medical University of Lodz; Ethic Approval/Registration Numbers: RNN/83/13/KE, RNN/178/16/KE. The patients/participants provided their written informed consent to participate in this study.

## Author contributions

MG contributed to data collection, analysed imaging, designed and conceptualised the study, analysed and interpreted all data, and drafted the manuscript. KS designed and conceptualised the study, analysed and interpreted all data, drafted and revised the manuscript, was the guarantor for this study. All authors contributed to the article and approved the submitted version.

## Funding

This study was supported by a grant PRELUDIUM from the National Science Center, Cracow, Poland, grant number UMO-2014/15/N/NZ4/01704.

## Conflict of interest

MG has received a grant from the National Science Center. KS has received personal compensation for consulting from Biogen, Celgene, GeNeuro, Merck, Novartis, Polpharma, Sanofi, Roche, TG Therapeutics, and received research support from Merck and Roche.

## Publisher's note

All claims expressed in this article are solely those of the authors and do not necessarily represent those of their affiliated organizations, or those of the publisher, the editors and the reviewers. Any product that may be evaluated in this article, or claim that may be made by its manufacturer, is not guaranteed or endorsed by the publisher.
